# Vimentin epigenetic deregulation in Bladder Cancer associates with acquisition of invasive and metastatic phenotype through epithelial-to-mesenchymal transition

**DOI:** 10.7150/ijbs.77181

**Published:** 2023-01-01

**Authors:** Sara Monteiro-Reis, Vera Miranda-Gonçalves, Catarina Guimarães-Teixeira, Cláudia Martins-Lima, João Lobo, Diana Montezuma, Paula C. Dias, Helene Neyret-Kahn, Isabelle Bernard-Pierrot, Rui Henrique, Carmen Jerónimo

**Affiliations:** 1 Cancer Biology and Epigenetics Group, Research Center of IPO Porto (CI-IPOP) / RISE@CI-IPOP (Health Research Network), Portuguese Oncology Institute of Porto (IPO Porto) / Porto Comprehensive Cancer Centre (Porto.CCC), Rua Dr. António Bernardino de Almeida, 4200-072, Porto, Portugal.; 2 INEGI-LAETA, Faculty of Engineering, University of Porto, Campus FEUP, Rua Dr. Roberto Frias 400, 4600-465, Porto, Portugal.; 3 Department of Pathology and Molecular Immunology, School of Medicine and Biomedical Sciences-University of Porto (ICBAS-UP), Rua de Jorge Viterbo Ferreira 228, 4050-313, Porto, Portugal.; 4 Department of Pathology, Portuguese Oncology Institute of Porto / Porto Comprehensive Cancer Center (Porto.CCC), Rua Dr. António Bernardino de Almeida, 4200-072, Porto, Portugal.; 5 Institut Curie, UMR144, Centre de Recherche, 75005 Paris, France.

**Keywords:** Vimentin, Methylation, Histones posttranslational modifications, Bladder Cancer, EMT.

## Abstract

Bladder cancer (BlCa) is the ninth most common cancer worldwide, associated with significant morbidity and mortality. Thus, understand the biological mechanisms underlying tumour progression is of great clinical significance. Vimentin (VIM) is (over)expressed in several carcinomas, putatively in association with EMT. We have previously found that VIM promoter methylation accurately identified BlCa and VIM expression associated with unfavourable prognosis. Herein, we sought to investigate VIM expression regulation and its role in malignant transformation of BlCa.

Analysis of tissue samples disclosed higher VIM transcript, protein, and methylation levels in BlCa compared with normal urothelium. VIM protein and transcript levels significantly increased from non-muscle invasive (NMIBC) to muscle-invasive (MIBC) cases and to BlCa metastases. Inverse correlation between epithelial *CDH1* and *VIM*, and a positive correlation between mesenchymal *CDH2* and *VIM* were also observed. In BlCa cell lines, exposure to demethylating agent increased VIM protein, with concomitant decrease in *VIM* methylation. Moreover, exposure to histone deacetylases pan-inhibitor increased the deposit of active post-translational marks (PTMs) across *VIM* promoter. In primary normal urothelium cells, lower levels of active PTMs with concomitant higher levels of repressive marks deposit were observed. Finally, *VIM* knockdown in UMUC3 cell line increased epithelial-like features and decreased migration and invasion *in vitro*, decreasing tumour size and angiogenesis *in vivo*.

We demonstrated that *VIM* promoter is epigenetically regulated in normal and neoplastic urothelium, which determine a VIM switch associated with EMT and acquisition of invasive and metastatic properties. These findings might allow for development of new, epigenetic-based, therapeutic strategies for BlCa.

## Introduction

Bladder cancer (BlCa) is a leading cause of cancer-related morbidity and mortality, being the 9^th^ most incident tumour worldwide 1, 2. Urothelial cell carcinomas (UCCs) are the most common form of BlCa, arising along the urinary tract, mostly in the lower tract (bladder and urethra), but also in the upper tract (renal pelvis and ureters) 3-7. The gold-standard diagnostic techniques are mostly invasive and uncomfortable, which led us and others to develop non-invasive tests 8-13. We have previously shown that a biomarker panel (*VIM, GDF15* and *TMEFF2* promoter methylation) accurately detected both lower and upper tract UCC in urine, and, more recently, that a multiplex panel (VIM and miR663a promoter methylation) accurately discriminated BlCa from inflammatory bladder conditions 8, 9, 11. From these studies, Vimentin (VIM) promoter methylation surfaced as the most promising biomarker, detecting alone most samples, as reported for other malignancies 14-19. Additionally, survival analysis showed that lower VIM promoter methylation levels independently predicted for poor disease-specific survival in upper tract urothelial carcinoma (UTUC) patients 9. Similar findings have been previously reported in cell lines of other epithelial tumours, in which VIM expression/overexpression was associated with increased tumour growth, invasion and poor prognosis 20, 21. Indeed, VIM is an intermediate filament characteristic of mesenchymal cells, usually not expressed in most normal epithelia (including urothelium) and epithelial tumours 22.

Epithelial-to-mesenchymal transition (EMT) is a multistep process through which epithelial cells develop mesenchymal characteristics, such as motility and invasive properties 23, 24. *In vitro* and *in vivo* studies indicate that EMT is associated with cancer cell invasion and metastasis in various malignancies 25-28. Remarkably, EMT is a reversible phenomenon, as cells may return to their epithelial phenotype in a process known as mesenchymal-to-epithelial transition (MET). Changes in expression of various molecular markers have been associated with EMT, including cadherin family and transcriptional repressors Zeb-1 and Zeb-2, Twist, Snail, Slug 29, 30. *VIM* is usually upregulated in cells undergoing EMT, a feature that has been implicated both normal in development and neoplastic progression 31. Nonetheless, the role of vimentin in EMT needs further clarification, and it is not clear how *VIM* expression is fine-tuned from its absence in normal cells to its (over)expression in invasive carcinoma cells. Moreover, the discovery of the molecular mechanisms that lead to this "switch" of VIM expression might identify novel therapeutic agents aiming to prevent cancer progression and metastization.

Epigenetic mechanisms, including DNA methylation and histone post-translational modifications (PTMs) dictate gene expression regulation, are reversible and its deregulation is common in cancer, 32, 33. Considering our previous observations on *VIM* promoter methylation in BlCa and its association with disease aggressiveness, we sought to characterize in depth the epigenetic mechanisms putatively responsible for VIM switch in this tumour model and ascertain the relevance of *VIM* expression deregulation for BlCa progression.

## Results

### Protein, transcript, and methylation analysis of VIM in bladder cancer tissues

**Figure 1:**
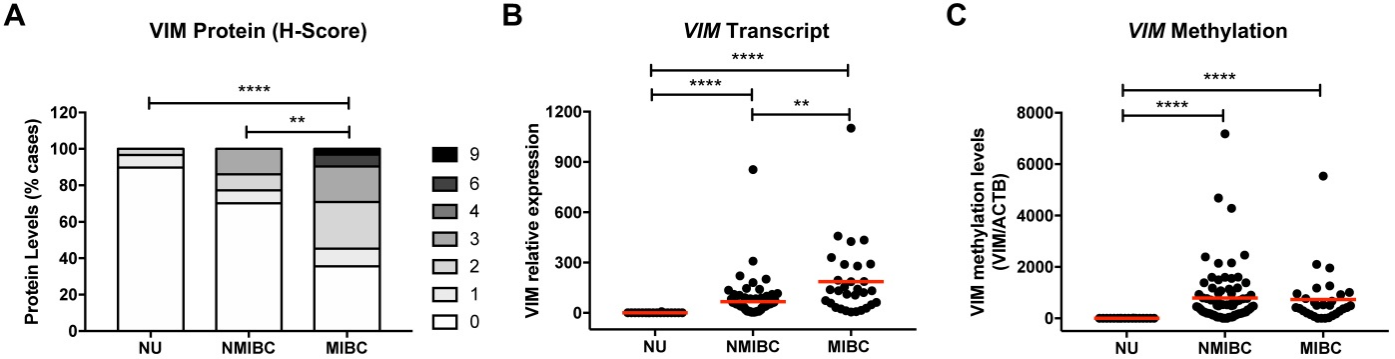
** VIM expression and methylation in normal urothelium and bladder cancer tissues. (A)** VIM immunohistochemistry results for normal urothelium (NU), non-muscle-invasive bladder cancer (NMIBC) tissues and muscle-invasive bladder cancer (MIBC) tissues, regarding the calculated immunoscore. **(B)** *VIM* transcript levels for NU, NMIBC and MIBC tissue samples by RT-qPCR. **(C)** *VIM* promoter methylation levels for NU, NMIBC and MIBC tissue samples by qMSP. *p<0.05, **p<0.01, ***p<0.001 and ****p<0.0001.

Immunohistochemistry and RT-qPCR analysis of VIM in NB tissue samples demonstrated that protein and transcript levels were low or undetectable (Figure 1A and 1B). Moreover, in the same samples, no methylation levels were detected at the *VIM* promoter (Figure 1C). BlCa cases disclosed higher VIM transcript, protein, and methylation levels, compared with NB (Figure 1). Importantly, VIM protein and transcript levels were significantly increased in MIBC compared to NMIBC, although no significant difference was apparent concerning *VIM* promoter methylation levels (Figure 1). Comparatively to primary BlCa tissue samples, metastatic BlCa tissues disclosed a significant increase in VIM protein levels and a concomitant decrease in *VIM* promoter methylation (Figure S1).

### Association between Cadherins and Vimentin expression in Bladder cancer tissues

To ascertain whether the increase of VIM expression in more invasive cases was related with EMT, *CDH1*, *CDH2* and *CDH3* (which encode for ECAD, NCAD and PCAD, respectively) transcript levels were first assessed in the same BlCa tissue samples. As expected, a significant decrease in *CDH1* (the epithelial cadherin) and a concomitant increase in *CDH2* (the mesenchymal cadherin) transcript levels were observed in MIBC, compared to NMIBC (Figure S2A and B). Moreover, no significant differences were observed for *CDH3* (Figure S2C). When performing correlation coefficient analysis, significant weak inverse correlations between *CDH1* and *CDH2*, and *CDH1* and *VIM* expression levels were found, whereas a significant weak positive correlation was depicted between *CDH1* and *CDH3* and *CDH2* and *VIM* transcript levels (Table S1; Figure S2D).

### Modulation of Vimentin methylation in BlCa cell lines and its impact in expression

**Figure 2:**
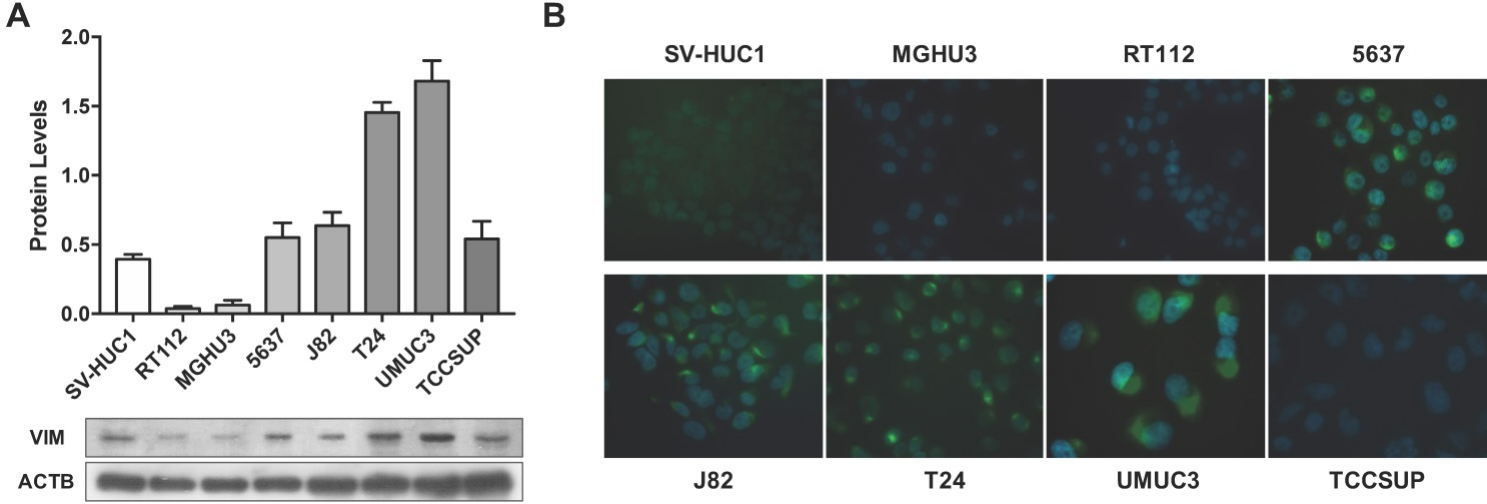
** VIM expression in bladder cancer cell lines. (A)** Expression of VIM protein in bladder cancer cell lines by Western blot; results are representative of three independent experiments with mean±SD. **(B)** Representative images of VIM protein localization in bladder cancer cell lines by immunofluorescence.

**Figure 3:**
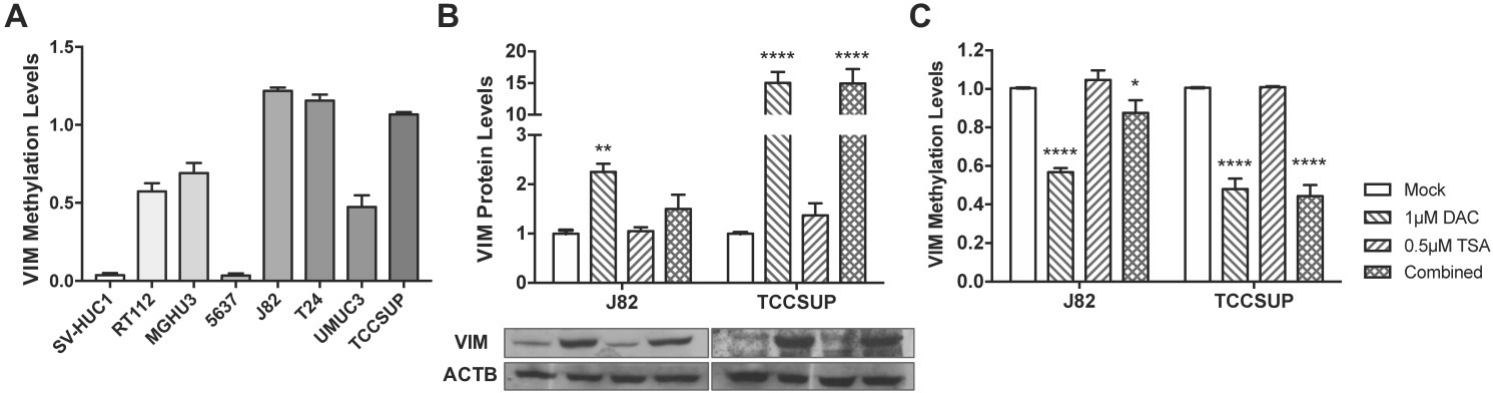
***VIM* methylation modulation in bladder cancer cell lines. (A)** *VIM* promoter methylation levels in bladder cancer cell lines by qMSP. **(B)** Expression of VIM protein in J82 and TCCSUP bladder cancer cell lines by Western blot, after treatment with epigenetic modulating drugs. **(C)** *VIM* promoter methylation levels in J82 and TCCSUP bladder cancer cell lines by qMSP, after treatment with epigenetic modulating drugs.

Considering the previous results in tissue samples, we investigated whether promoter methylation might regulate *VIM* expression in BlCa, using *in vitro* models. Firstly, VIM protein levels were assessed by WB and IF in the available BlCa and normal bladder immortalized cell lines, to determine which cells disclosed the lowest VIM expression levels (Figure 2). Then, using the same cell lines, *VIM* promoter methylation was assessed, and J82 and TCCSUP were chosen to perform subsequent treatment with epigenetic drugs DAC and TSA, as these two cell lines disclosed low VIM expression with concomitant high *VIM* promoter methylation levels (Figure 2A and 3A).

In J82 and TCCSUP cells, 1μM DAC exposure significantly increased VIM protein levels (p<0.01 and p<0.0001, respectively; Figure 3B), and the same was observed in TCCSUP cells treated with both drugs (p<0.001), compared to mock cells. Cell lines treated with TSA only, did not disclose variation of VIM expression levels. When *VIM* methylation levels were assessed in the same treated cell lines, for the same conditions, lower methylation levels were observed in cells treated with DAC and/or DAC+TSA (Figure 3C), supporting a role for promoter methylation in *VIM* expression regulation in BlCa cells.

### Regulation of VIM expression by histone posttranslational modifications

**Figure 4:**
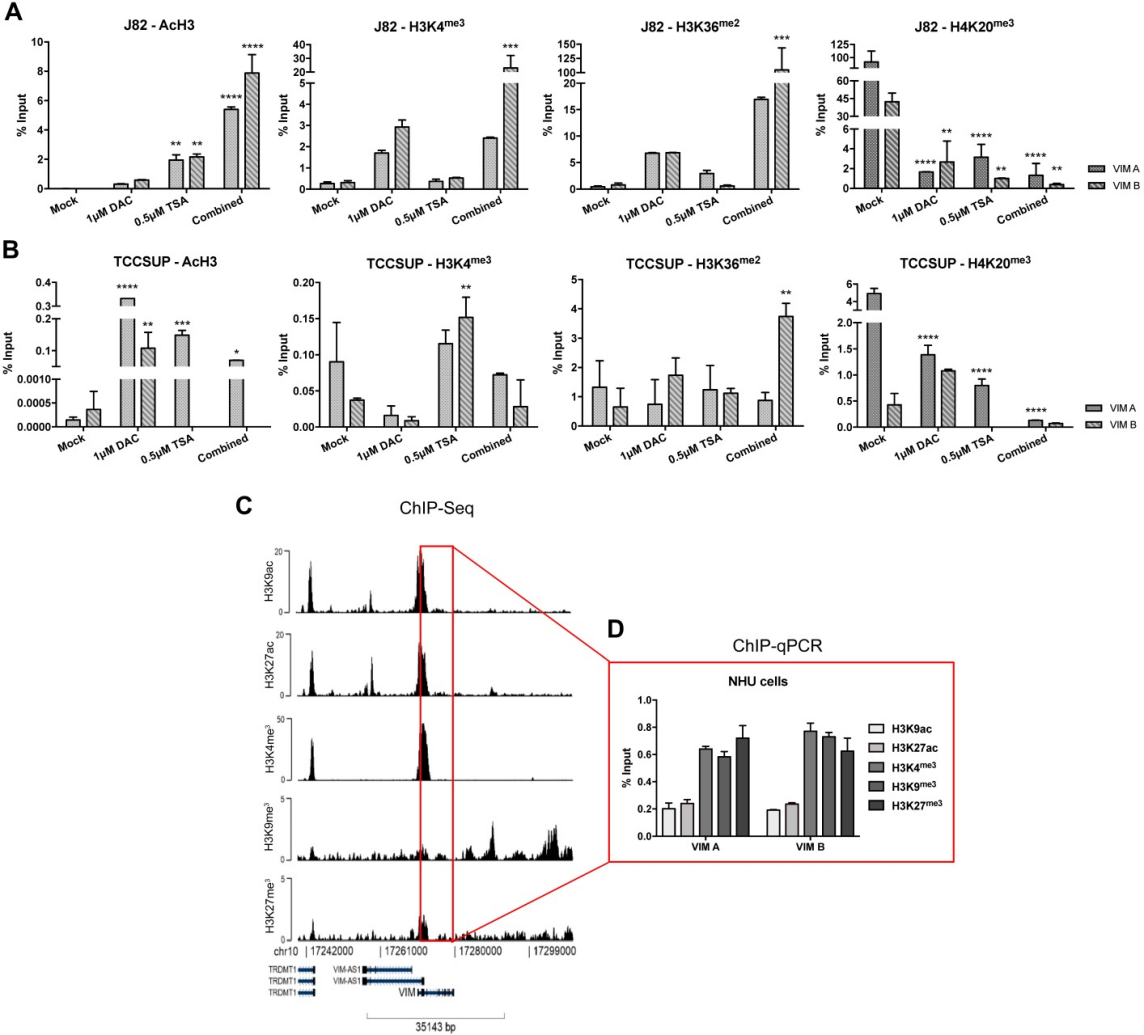
ChIP-qPCR results for **(A)** J82 and **(B)** TCCSUP cell line concerning acH3, H3K4me3, H3K36me2 and H4K20me3 histones marks across VIM promoter, after treatment with epigenetic modulating drugs. **(C)** ChIP-seq representative results for NHU cells across VIM gene promoter and body. **(D)** ChIP-qPCR results for NHU cells concerning H3K9ac, H3K27ac, H3K4me3, H3K9me3 and H3K27me3 histones marks across VIM promoter. Results are normalized with the input of total sonicated chromatin.

**Figure 5:**
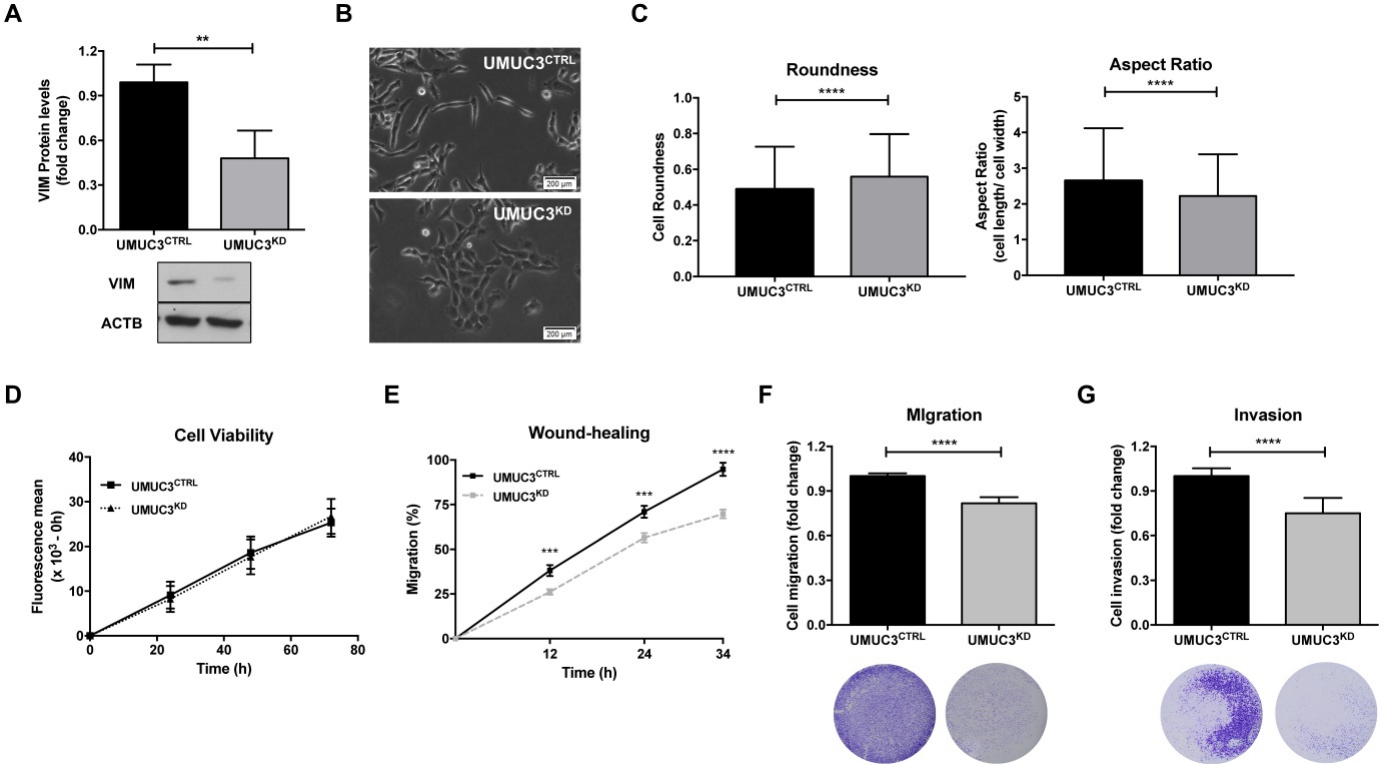
** VIM downregulation impairs migration and invasiveness in bladder cancer cells**. **(A)** Protein expression of VIM in UMUC3^CTRL^ and UMUC3^KD^ cells by Western Blot.** (B)** Representative phase-contrast images of UMUC3^CTRL^ and UMUC3^KD^ cells. **(C)** Cell morphometric parameters - roundness and aspect ratio (cell length/ cell width) - analysis in UMUC3^CTRL^ and UMUC3^KD^ cells. Effect of VIM knockdown in UMUC3^CTRL^ and UMUC3^KD^ cells at **(D)** cell viability by Resazurin assay, **(E)** cell migration by wound-healing assay and by **(F)** polycarbonate insert chambers, and **(G)** cell invasion by BD Biocoat Matrigel Invasion Chambers; **p<0.01, ***p<0.001 and ****p<0.001; results are representative of three independent experiments with mean±SD, each of them in triplicates.

To determine whether histones PTMs might also contribute to *VIM* expression regulation, ChIP-qPCR analysis for known repressive and activating PTMs was performed in the same cell lines exposed to epigenetic drugs. For TCCSUP cell line, treatment with TSA (alone or in combination) significantly increased the deposit of AcH3, H3K4^me3^ and H3K36^me2^ active marks across *VIM* promoter (Figure 4B), corroborating the previously observed increase in protein levels for the same conditions (Figure 3B). Although no significant increment in VIM protein levels was previously observed in J82 cells exposed to TSA or DAC+TSA, similar deposit of active marks across *VIM* promoter was depicted by ChIP (Figure 5A). For both cell lines, a decrease in H4K20^me3^ repressive mark was also observed, in all treatment conditions (Figure 4A and B).

Because the previous results suggested that PTMs might play a role in *VIM* regulation and considering that *VIM* methylation was not detected in normal urothelial cells, we hypothesized whether PTMs might be involved in *VIM* downregulation in normal urothelium. Thus, ChIP-qPCR for known repressive and activating PTMs was performed in SVHUC1 immortalized (normal) urothelial cell line. Interestingly, high levels of two active marks - H3K27ac and H3K36^me2^ - and lower levels of two repressive marks - H3K9^me3^ and H3K27^me3^ - were detected across *VIM* promoter in this cell line (Figure S3), which may explain the low/moderate VIM protein levels previously detected in these cells (Figure 3). Subsequently, ChIP-seq was performed in a primary normal human urothelial cell line, followed by validation with ChIP-qPCR for the most relevant PTMs. Remarkably, lower levels of active PTMs - H3K9ac and H3K27ac - with concomitant higher levels of repressive marks deposit - H3K4^me3^, H3K9^me3^ and H3K27^me3^ - was observed, suggesting that histones PTMs are indeed important for the repression of *VIM* expression in normal urothelium (Figure 4C and D).

### Phenotypic impact of *VIM* modulation in BlCa cells

To assess the phenotypic impact of *VIM* deregulation in BlCa, UMUC3 cells were selected to perform *VIM* forced knockdown, as this cell line disclosed the highest protein expression (Figure 2). After CRISPR/Cas9 mediated gene knockdown, a significant reduction in VIM protein levels (approximately 60%) was achieved (p = 0.0022; Figure 5A).

Morphological alterations were also observed in UMUC3^KD^ cells, displaying cobblestone (i.e., epithelial-like) features with increased cell-cell contacts, whereas UMUC3^CTRL^ cells depicted an elongated (mesenchymal-like) shape (Figure 5B). The observed morphological differences were corroborated by morphometric analysis: UMUC3^KD^ cells disclosed significantly higher roundness and decreased (length/ width) aspect ratio parameter (Figure 5C).

Although no significant differences in cell viability (Figure 5D) were found between UMUC3^KD^ and UMUC3^CTRL^, cell invasion and migration were decreased in *VIM* knockdown cells (Figure 5E, F and G).

### *VIM* knockdown effect in *In vivo* tumor formation

**Figure 6:**
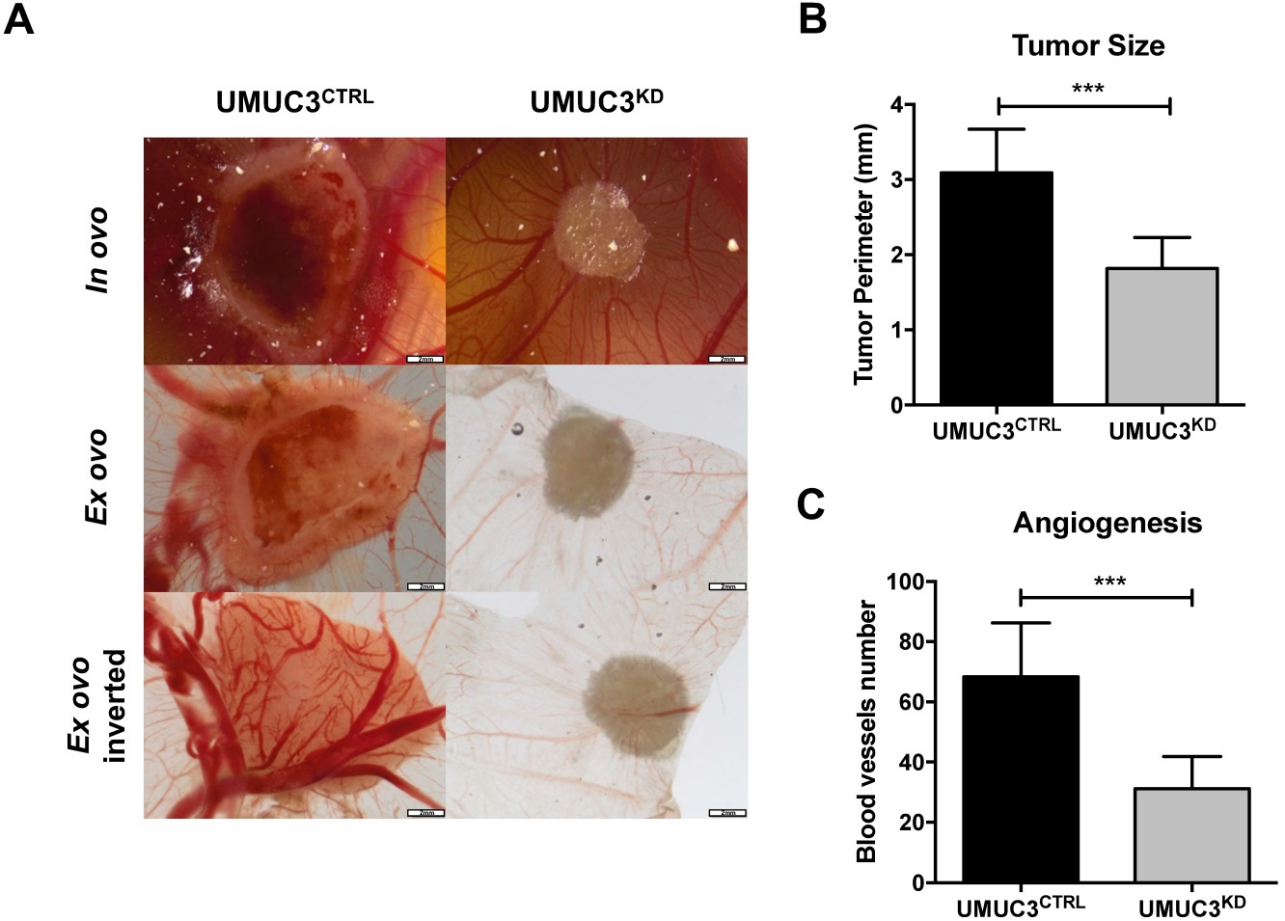
** Knockdown of VIM attenuates the malignant phenotype in vivo**. **(A)** Macroscopic view of tumor formation (*in ovo* and *ex ovo*) and neo-angiogenesis UMUC3^CTRL^ and UMUC3^KD^ experimental conditions. Distribution of macroscopic **(B)** tumor size and **(C)** number of peri-tumor vessels in UMUC3^CTRL^ and UMUC3^KD^ experimental conditions; ***p<0.001.

Overall, cells harboring *VIM* knockdown presented significantly reduced microtumors' size in CAM, comparing with those originated from UMUC3^CTRL^cells (p = 0.0003, Figure 6A and B). These results were paralleled by the decreased number of formed blood vessels (p = 0.0003, Figure 6A and C).

## Discussion

Although clinical management and molecular characterization of BlCa have progressed considerably over the past few years, it remains a foremost health concern, due to high incidence and recurrence rates, entailing significant patient morbidity and economic burden. Thus, identification of more accurate biomarkers which might perfect disease monitoring and prognostication are deemed to have significant clinical and societal impact. In our previous studies, *VIM* surfaced as potentially useful BlCa biomarker, especially quantitative promoter methylation, which disclosed diagnostic and prognostic value 8, 9, 11. Herein, we sought to extend those findings, looking for a putative epigenetic regulation of VIM expression, impacting on BlCa aggressiveness.

*VIM* expression and methylation analysis of normal and cancerous (both primary and metastatic) urothelial tissues, confirmed our previous results concerning the specificity of *VIM* promoter methylation in BlCa vs. normal urothelium 8, 9, 11. Nonetheless, normal urothelium disclosed very low or even absent levels of VIM expression, whereas BlCa tissues showed increased expression. Moreover, metastatic lesions disclosed higher VIM expression and lower methylation levels, compared to primary BlCa. VIM is an intermediate filament, characteristic of cells with mesenchymal phenotype, not expressed in most normal epithelia (including urothelium) nor carcinomas 34. However, VIM de-novo expression or overexpression has been reported in various epithelial cancers, including those of prostate, breast, and lung, associating with increased tumor growth, invasion and poor prognosis 20, 21, 35. Those findings have been related with EMT, a biological process underlying invasive and metastatic properties of malignant epithelial cells. Thus, VIM expression pattern in NB, primary and metastatic BlCa is consistent with the acquisition of EMT traits by invasive and metastatic neoplastic urothelial cells. As to *VIM* promoter methylation, our findings suggest that it develops during neoplastic transformation, eventually as a bystander alteration in primary tumors, but modulated in secondary lesions to accomplish more effective EMT in metastatic cells.

Although *VIM* promoter methylation and expression patterns in tissues were not fully consistent with a regulatory role, exposure of BlCa cell lines to demethylating agent DAC resulted in increased VIM expression, confirming that, indeed, *VIM* promoter methylation is involved in gene expression regulation. Nonetheless, additional regulatory mechanisms are required and characterization of histone PTMs across *VIM* promoter in those cell lines uncovered the importance of those epigenetic regulatory mechanisms in the same gene. Remarkably, in a primary normal urothelial cell line, ChIP experiments disclosed deposit of repressive marks at the expense of active PTMs, suggesting that this mechanism (but not promoter methylation) is determinant for maintaining low VIM levels in those normal cells. Thus, we may conclude that both promoter methylation and histone PTMs have important regulatory functions in VIM expression in urothelial cells, with histone PMTs playing the foremost role in *VIM* silencing in normal cells, whereas they act in concert with promoter methylation in cancerous cells to modulate *VIM* expression according to the intensity of invasive and metastatic behavior, orchestrated through EMT.

This hypothesis is further supported by the results of *CDH1* and *CDH2* expression in BlCa tissues and its significant, although weak, correlation with *VIM* transcript levels, which disclosed *CDH1* downregulation and *CDH2* and *VIM* upregulation in MIBC compared to NMIBC, a pattern which is consistent with ongoing EMT in invasive urothelial cells. The impact of *VIM* expression in cell phenotype was further demonstrated, as malignant urothelial cells with downregulated *VIM* expression (re)acquired a more epithelial-like morphology (denoting reversion of EMT, or MET) and impaired cell motility, decreasing both cell migration and cell invasion capabilities, although it did not affect cell viability. Importantly, the absence of VIM expression significantly diminished tumor growth in CAM, as well as of neoformed blood vessels. Thus, to the best of our knowledge, this is the first study characterizing the “Vimentin switch” occurring in urothelial carcinogenesis, also demonstrating its biological relevance concerning migratory capabilities of neoplastic cells and tumor formation. Interestingly, we had previously shown that patients with NMIBC disclosing higher VIM expression endured poorer disease-free survival, with increased expression depicted along the sequence NB-NMIBC-MIBC-Metastases 36. Furthermore, VIM expression has been also associated with BlCa grade and stage. Indeed, Baumgart et al. found that VIM expression was mainly detected in invasive BlCa (31% in MIBC *vs.* 7% in NMIBC) and positively associated with tumor grade and stage, whereas Paliwal et al. found that VIM immunoexpression correlates with BlCa stage and grade 37, 38. Thus, VIM expression is closely associated with invasive and metastatic properties of malignant urothelial cells and may serve as a marker of disease aggressiveness.

A major limitation of our study is the limited number of cases analyzed. Thus, a larger and, ideally, multicenter study, is required to validate our findings. Moreover, the assessment of non-coding RNAs function in VIM regulation would further enlighten its implication in bladder carcinogenesis. Nonetheless, it should be emphasized that our findings are coherent, confirming and extending previous observations from our research team and others, concerning the role of VIM in BlCa progression.

In conclusion, our findings, based on the analysis of urothelial tissue samples and modulated cell lines, further support that VIM is implicated in acquisition of malignant urothelial phenotype. We propose a model in which VIM expression is repressed in normal urothelium, mostly through histone PTMs, whereas *VIM* promoter methylation is acquired during neoplastic transformation, most likely as a passenger alteration. Then, neoplastic cells undergo EMT with increased VIM expression achieved through decreased promoter methylation in concert with active histone PTMs acquisition, enabling tumor growth and favoring local invasion and systemic dissemination, fostering disease progression.

## Materials and methods

### Patients and Samples

Patients (n=108) with primary BlCa, treated with transurethral resection (TUR) or radical cystectomy, between 1991 and 2011 at Portuguese Oncology Institute of Porto (IPO Porto), with available frozen and formalin-fixed paraffin-embedded (FFPE) tissue at the Department of Pathology, were included in this study. A set of 36 morphologically normal bladder mucosa (NB) tissue samples was obtained from BlCa-free individuals (prostate cancer patients submitted to radical prostatectomy) and served as controls. Additionally, a set of FFPE tissue samples from 28 metastasis (Met) of BlCa patients were also collected. All primary specimens were fresh-frozen at -80ºC and subsequently cut in a cryostat for confirmation of representativity and nucleic acid extraction. From each specimen, fragments were collected, formalin-fixed, and paraffin embedded for routine histopathological examination, including grading and pathological staging, by a dedicated uropathologist 39. Relevant clinical data was collected from clinical charts (Table 1). Patients and controls were enrolled after informed consent. This study was approved by the institutional review board (Comissão de Ética para a Saúde) of IPO Porto (CES015-2016). The CIT cohort (*Carte d'Identité des Tumeurs*) including 98 NMIBC, 101 MIBC and 4 normal urothelium was previously described in Rebouissou et al. and Biton et al., and the TCGA cohort including 412 MIBC in Robertson et al., 2017 40-42.

### Cell lines

BlCa cell lines (RT112, MGHU3, 5637, J82, T24, UMUC3 and TCCSUP) and normal bladder cell line SV-HUC1, available at our lab, were selected for this study. All cell lines were purchased from ATCC and grown using recommended medium (Biochrom-Merck, Berlin, Germany) supplemented with 10% fetal bovine serum (FBS, Biochrom) and 1% penicillin/streptomycin (GBICO, Invitrogen) at 37ºC and 5% CO_2_. Additionally, a primary cell line culture from normal human urothelium (NHU), kindly provided by Dr. Isabelle Pierrot (Institut Curie, Paris, France) was also cultured for ChIP-qPCR assays. Establishment and culture conditions are described in Neyret-Kahn et al 43.

Mycoplasma testing was regularly performed (every two weeks) in all cell lines, using TaKaRa PCR Mycoplasma Detection Set (Clontech Laboratories, Mountain View, CA, EUA).

### RNA isolation, Real-Time Quantitative PCR (RT-qPCR) and analysis of transcriptomic data

RNA was extracted from tissues using TRIzol^®^ (Invitrogen, Carlsbad, CA, USA), according to manufacturer's instructions. cDNA synthesis was performed using the High Capacity cDNA Reverse Transcription Kit (Applied Biosystems^®^, Foster City, CA, USA), according to manufacturer's instructions. Target genes transcript levels were quantified by RT-qPCR. Expression levels were evaluated using either 4.5µL of diluted cDNA, 5µL of TaqMan^®^ Universal PCR Master Mix No AmpErase^®^ UNG (Applied Biosystems^®^) and 0.5 µL of specific TaqMan^®^ Gene Expression Assay (*HPRT1* - Ref. ID Hs01003267_m1, *VIM* - Ref. ID Hs00185584_m1), or using Xpert Fast SYBER Mastermix Blue (GRiSP Research Solutions, Porto, Portugal) with specific primers for each target and reference genes, as described in Table S2. Each sample was run in triplicate under the following RT-qPCR conditions: 2 minutes at 50ºC, followed by enzyme activation for 10 minutes at 95ºC, and 45 cycles which included a denaturation stage at 95ºC for 15 seconds and an extending stage at 60ºC for 60 seconds. *HPRT* was used as reference gene for normalization. Relative expression of target genes tested in each sample was determined as: [Gene Expression Level = (Gene Mean Quantity / Reference Gene Mean Quantity) x 1000]. For the CIT cohort, VIM, CDH1, CDH2 and CDH3 expression levels were extracted from available transcriptomic Affymetrix U133Plus2.0 data 40, 41. An EMT score for each tumor was also determined using these data and a previously published signature, combining 206 epithelial genes and 132 mesenchymal genes 44. EMT scores were calculated by subtracting the mean log2 normalized expression of the epithelial genes from that of the mesenchymal genes in each sample. We also analyzed VIM expression levels in 4 cell lines (MGHU3, TCCSUP, RT112 and UMUC3) from available Affymetrix transcriptomic data 40.

### DNA isolation, Bisulfite Modification and Quantitative Methylation Specific PCR (qMSP) Analysis

DNA was extracted from frozen BlCa and NB tissues and cell lines using a standard phenol-chloroform protocol 45, and its concentration determined using a Qubit 3 Fluorometer (Thermo Fisher Scientific, Waltham, MA, USA). Bisulfite modification was performed through sodium bisulfite, using the EZ DNA Methylation-Gold™ Kit (Zymo Research, Irvine, CA, USA), according to manufacturer's protocol. For this, 1000ng of DNA were converted. Quantitative methylation levels were performed using Xpert Fast Probe Master Mix (GRiSP, Porto, Portugal), in 96-well plates using an Applied Biosystems 7500 Sequence Detector (Perkin Elmer, Waltham, CA, USA), with Beta-Actin (ACTB) as internal reference gene for normalization. Primer and probe sequences were designed using Methyl Primer Express 1.0 and purchased from Sigma-Aldrich (St. Louis, MO, USA) (Table S2). Additionally, six serial dilutions (dilution factor of 5 ×) of a fully methylated bisulphite modified universal DNA control were included in each plate to generate a standard curve. In each sample and for each gene, the relative DNA methylation levels were determined using the following formula: ((target gene/ACTB) ×1000). A run was considered valid when previously reported criteria were met 8. For TCGA cohort, 450k methylation array data available for 386 MIBC were analysed using Wanderer tool with a focus on VIM locus 46.

### Immunohistochemistry

Immunohistochemistry was performed using the Novolink™ Max Polymer Detection System (Leica Biosystems, Wetzlar, Germany]. Three-μm thick tissues sections from formalin-fixed and paraffin-embedded BlCa, NU and Met samples were cut, deparaffinized and rehydrated. Antigen retrieval was accomplished by microwaving the specimens at 800W for 10 minutes in 10mM sodium citrate buffer, pH=6. After, endogenous peroxidase activity was blocked, primary monoclonal antibody for VIM (NCL-L-VIM-V9, Leica) was used in 1:100 dilution, and incubated at room-temperature (RT) for one hour. Then, 3, 3′-diaminobenzidine (Sigma-Aldrich™) was used as chromogen for visualization and slides were mounted with Entellan^®^ (Merck-Millipore, Burlington, MA, EUA). Normal tonsil tissue, showing intense VIM immunoreactivity was used as positive control. VIM immunoexpression was evaluated by a dedicated uropathologist and cases were classified using a semi-quantitative scale for both staining intensity (0—no staining; 1—intensity lower than normal urothelium; 2—intensity equal to normal urothelium; 3—intensity higher than normal urothelium) and percentage of positive cells (0—< 10%; 1—10-33%; 2—33-67%; 3—>67%), in each tumor. Staining intensity and percentage of positive cell results were then combined into a single score (Score S = staining intensity x percentage of positive cells) assigned to each tumor.

### Cell lines treatment with epigenetic drugs

Cell lines TCCSUP and J82 were grown, until 20 to 30% confluence was reached, in 75cm^3^ cell culture flasks. Then, media containing the corresponding drug(s) - a pharmacologic inhibitor of DNMTs, 5-aza-2-deoxycytidine (DAC) (Sigma-Aldrich®, Germany) and/or a pan-inhibitor of HDAC, Trichostatin A (TSA) (Sigma-Aldrich®, Germany) - were added at 1μM and 0.5μM concentrations, respectively. Culture medium and appropriate drug(s) were renewed every 24h, on a total of 72h. Mock cells served as controls as they were submitted to the medium change procedure but were only exposed to drug(s) vehicle(s). All treatment experiments were done in triplicate. On the fourth day of the treatment schedule, cells were harvested by trypsinization and centrifuged. Then, either pellets were washed in PBS 1x and stored at -80^o^C for DNA extraction or were immediately processed for protein extraction or ChIP analysis.

### VIM gene knockdown

*VIM* knockdown was performed through CRISPR/Cas9 system, delivered to cells through a plasmid including a specific guide RNA (gRNA) sequence targeting *VIM*, and puromycin-resistance gene (GenScript, Piscataway, NJ, EUA). Briefly, UMUC3 cells were seeded in 24 well/plates and let to grow until approximately 85% confluency. Then, transfection was performed using Lipofectamine 3000 reagent (Invitrogen, USA), and cells were incubated with the plasmid for 48h. Subsequently, 1µg/mL of puromycin dihydrochloride (Clontech Laboratories) was added to select stably transfected cells (UMUC3^KD^). Control cells were generated by transfecting UMUC3 cell line with an empty gRNA construct (GenScript) following the same above-mentioned transfection and selection conditions (UMUC3^CTRL^). Cells were then grown until confluence, and passed at least two times, until protein extraction and subsequent western blot analysis for VIM expression.

### Protein extraction and Western Blot (WB) analysis

SVHUC1, UMUC3^KD^ and UMUC3^CTRL^ cell lines were grown until 80% confluence and homogenized in Kinexus lysis buffer supplemented with proteases inhibitors cocktail. Then, cells were sonicated for 5 cycles of 30 seconds ON and 30 seconds OFF (Bioruptorâ, Diagenode, Liège, Belgium). After centrifugation, the supernatant was collected, and total protein was quantified according to the Pierce BCA Protein Assay Kit (Thermo Fisher Scientific Inc.), according to the manufacture procedure.

Thirty µg total protein were separated in 10% polyacrylamide gel by SDS-PAGE and transferred onto an immunoblot PVDF membrane (Bio-Rad Laboratories, Hercules, CA, USA) in a 25mM Tris-base/ glycine buffer using a Trans-Blot Turbo Transfer system (Bio-Rad Laboratories). Membranes were blocked with 5% bovine serum albumin (BSA) in TBS/0.1% Tween (TBS/T pH=7.6) for 2h at RT. After incubation with VIM primary antibody (NCL-L-VIM-V9, Leica), membranes were washed in TBS/T and incubated with secondary antibody coupled with horseradish peroxidase, for 1h at RT. Binding was visualized by chemiluminescence (Clarity WB ECL substrate, Bio-Rad) and quantification was performed using band densitometry analysis from the ImageJ software (version 1.6.1, National Institutes of Health). β-Actin (A1978, Sigma-Aldrich) was used as loading control.

### Immunofluorescence (IF) and Morphometric analysis

SVHUC1, UMUC3^KD^ and UMUC3^CTRL^ cell lines were seeded on cover slips at 20,000 cells/well, overnight. Briefly, cells were fixed in methanol during 10min and then blocked with 5% bovine serum albumin (BSA) during 30min. After overnight VIM (1:150, #3195, Cell Signaling Technology) incubation at room temperature, cells were incubated with secondary antibody anti-rabbit IgG-TRITC (1:500, T6778, Sigma-Aldrich) during 1h at RT. Finally, after washing in 1X PBS, cells were stained with 4',6-diamidino-2-phenylindole (DAPI) (AR1176, BOSTER Biological Technologies) in mounting medium. Pictures were taken in a fluorescence microscope Olympus IX51 with a digital camera Olympus XM10 using CellSens software (Olympus Corporation, Shinjuku, Japan). Cell morphometric parameters - roundness and aspect ratio (cell length/ cell width) - were calculated for UMUC3^KD^ and UMUC3^CTRL^ cells using the ImageJ software, in three independent experiments.

### Chromatin Immunoprecipitation (ChIP)

Chromatin Immunoprecipitation (ChIP) analysis was performed in J82, TCCSUP, SVHUC1 and NHU cells, following a previously published protocol 47. ChIP-grade antibodies for specific PTMs (Table S3), positive control (RNA polymerase II) and negative control (mouse IgG), were used at assay dependent concentration. For qPCR, two pairs of primers for *VIM* promoter were designed, both for ~325bp (VIM A) before TSS (F—5'- TAGTGAGCAGGAGAAAGCACAG-3', R-5'-AAAGACAGGACATGGAGGATGT-3') and for ~600bp (VIM B) before TSS (F—5'-CTGAACTGATACAGTGGCAAGTGA-3', R—5'-TCAGGATATGCATGCCAAAG-3'). RT-qPCR was performed as mentioned above, and the relative amount of promoter DNA was normalized using Input Percent method. ChIPseq was performed in NHU proliferating cells and analyzed as previously described in Neyret-Kahn et al 43.

### Cell viability assay

Resazurin viability assay, (Canvax Biotech, Córdoba, Spain) was performed for UMUC3^KD^ and UMUC3^CTRL^ cells at 24h, 48h and 72h. Briefly, cells were plated into 96-well plates in medium at density of 3000 cells/well and incubated overnight, at 37ºC in 5% CO_2_. For each time point, cells were incubated for 3 hours at 37ºC with 1:10 Resazurin solution in culture medium. Then, the solution was removed, and fluorescence measurement was carried out at wavelength 530-570 nm excitation and 585-590 nm emission in a microplate reader (Fluostar Omega, BMG Labtech, Germany). The ODs obtained for each time point were all normalized for the 0 hours-time point. All experiments were performed in biological triplicates, each with experimental triplicates.

### Wound healing assay

UMUC3^KD^ and UMUC3^CTRL^ cells were seeded in 6-well plate at a density of 7.5x10^5^ cell/well and allowed reach confluence at 37ºC, 5% CO_2_. Then, a “wound” was made by manual scratching with a 200 µL pipette tip and cells were gently washed with 1X PBS. The ''wounded'' areas were photographed in specific wound sites (two sites for each wound) at 40x magnification using an Olympus IX51 inverted microscope equipped with an Olympus XM10 Digital Camera System every 12h until wound closure. The relative migration distance (5 measures by wound) was calculated with the following formula: relative migration distance (%) = (A-B)/C x100, where A is the width of cell wound at 0h incubation, B is the width of cell wound after specific hours of incubation, and C is the width mean of cell wound for 0h of incubation. For relative migration distance, the results were analyzed using the beWound-Cell Migration Tool (Version 1.5) (developed by A.H.J. Moreira, S. Queirós and J.L. Vilaça, Biomedical Engineering Solutions Research Group, Life and Health Sciences Research Institute- University of Minho; available at http://www. besurg.com/sites/default/files/beWoundApp.zip). At least three independent experiments were performed.

### Migration and Invasion assays with insert chambers

Migration and invasion capacities were assessed for UMUC3^KD^ and UMUC3^CTRL^ cells using polycarbonate insert chambers (Thermo Fisher Scientific) and BD BioCoat Matrigel Invasion Chambers (BD Biosciences), respectively. After rehydration of inserts in DMEM medium for 2 hours at 37ºC, cells were seeded at density of 4.5x10^4^ cells/insert and incubated 24h at 37ºC in 5% CO_2_. Then, non-migrating/non-invading cells were removed by swab and migrated/invaded cells were fixed with 4% PFA for 2 min and with cold methanol during 20min, followed by cell staining with Cristal Violet for 10min. Membranes were photographed in Olympus SZx16 stereomicroscope (10x magnification), and migrating/invading cells were counted using the Image J software (version 1.41; National Institutes of Health). At least three independent experiments were performed for each condition.

### Chorioallantoic membrane (CAM) Assay

Fresh fertilized eggs (PintoBar, Lda, Portugal) were incubated at 37 °C in a humidified environment. After 6 days of embryonic development, a window was opened into the eggshell under aseptic conditions. On day 10, UMUC3^KD^ or UMUC3^CTRL^ cells suspensions in growth factor-reduced Matrigel (BD Biosciences) were seeded on CAM. On day 17, microtumor images were obtained, and tumors were dissected, formalin fixed and included in paraffin. Relative perimeter *in ovo* was assessed using CellSens software (version V0116, Olympus). *Ex ovo* microphotographs were obtained for blood vessels counting, using Image J software.

### Statistical analysis

All statistical analyses were performed using IBM^®^ SPSS^®^ Statistic software version 23 (IBM-SPSS Inc., Chicago, IL, USA) and graphs were built using GraphPad Prim 7.0 (GraphPad Software Inc., La Jolla, CA, USA). Significance level was set at p<0.05, and Bonferroni's correction was used when appropriate. Mann-Whitney U test (MW) was used to test for differences in VIM expression or methylation levels between NB and BlCa, pathological stages of cases divided into Ta-1 (non-muscle invasive BlCa, NMIBC) and T2-4 (muscle invasive BlCa, MIBC), and patients' gender, and to assess differences in UMUC3^KD^ and UMUC3^CTRL^ conditions. Kruskall-Wallis test (KW) was performed to test for differences among more than two groups of samples then followed by MW test when appropriate. Spearman's rho was used to assess the correlation between VIM expression or methylation levels and age of the patients at diagnosis, and between *VIM* and *CDH1, CDH2* or *CDH3* transcript levels. Associations between clinical grade or pathological stage and immunoexpression results were assessed by chi-square or Fisher's exact test, and Somers' D directional measure was also computed.

Disease-specific and disease-free survival curves (Kaplan-Meier with log rank test) were computed for standard variables (tumor stage and grade) and for categorized *VIM* transcript and methylation levels. Moreover, the same analyses were also performed separately for NMIBC and MIBC cases. A Cox-regression model comprising all significant variables (univariable and multivariable model) was computed to assess the relative contribution of each variable to the follow-up status.

## Supplementary Material

Supplementary figures and tables.

## Figures and Tables

**Table 1:** Clinical and histopathological parameters of Bladder Cancer patients, and gender and age distribution of control set individuals.

Clinicopathological features	Bladder Cancer	Normal Urothelium
Patients, n	108	36
Gender, n (%)		
Males	78 (72)	23 (64)
Females	30 (28)	13 (36)
Median age, yrs (range)	69 (45-91)	63 (48-75)
Grade, n (%)		
Papillary, low-grade	37 (34)	n.a.
Papillary, high-grade	31 (29)	n.a.
Invasive, high-grade	40 (37)	n.a.
Pathological Stage, n (%)		
pTa/pT1 (NMIBC)	68 (63)	n.a.
pT2-4 (MIBC)	40 (37)	n.a.

(n - number; yrs - years; pT - pathological stage; NMIBC - non-muscle invasive bladder cancer; MIBC - muscle invasive bladder cancer).
