# Supplementary material for: Vimentin epigenetic deregulation in Bladder Cancer associates with acquisition of invasive and metastatic phenotype through epithelial-to-mesenchymal transition
Source: Int J Biol Sci. 2023 Jan 1;19(1):1–12. doi: 10.7150/ijbs.77181 (PMC9760433; doi:10.7150/ijbs.77181)
Supplement: Supplementary file 1 — Supplementary figures and tables. [file ijbsv19p0001s1.pdf]

Figure S1: *VIM* expression and methylation in normal urothelium, bladder cancer and bladder cancer metastasis. (A) VIM immunohistochemistry results for normal urothelium (NU), non-muscle-invasive bladder cancer (NMIBC), muscle-invasive bladder cancer (MIBC) and metastasis (Met) tissues regarding the calculated immunoscore. **(B)** Methylation levels for NU, NMIBC, MIBC and Metastasis tissue samples by qMSP. *$p<0.05$, **$p<0.01$, ***$p<0.001$ and ****$p<0.0001$.

Figure S2: Cadherins genes expression in bladder cancer tissues. Transcript levels of **(A)** *CDH1*, **(B)** *CDH2* and **(C)** *CDH3* in non-muscle-invasive bladder cancer (NMIBC) tissues and muscle-invasive bladder cancer (MIBC) tissues by RT-qPCR. **(D)** Graphical representation of transformed log data comparison of *VIM*, *CDH1*, *CDH2* and *CDH3* transcript levels in bladder cancer tissue samples. Transcript levels were obtained by RT-qPCR.

Figure S3: ChIP-qPCR for histone PTMs across *VIM* promoter, in SVHUC1 immortalized urothelial cell line. ChIP-qPCR results for H3K27Ac, H3K36me2, H3K9me3 and H3K27me3 across *VIM* promoter region. VIM A represents a region ~325bp before TSS, and VIM B a region ~600bp before TSS. Results are normalized with the input of total sonicated chromatin.

Figure S1

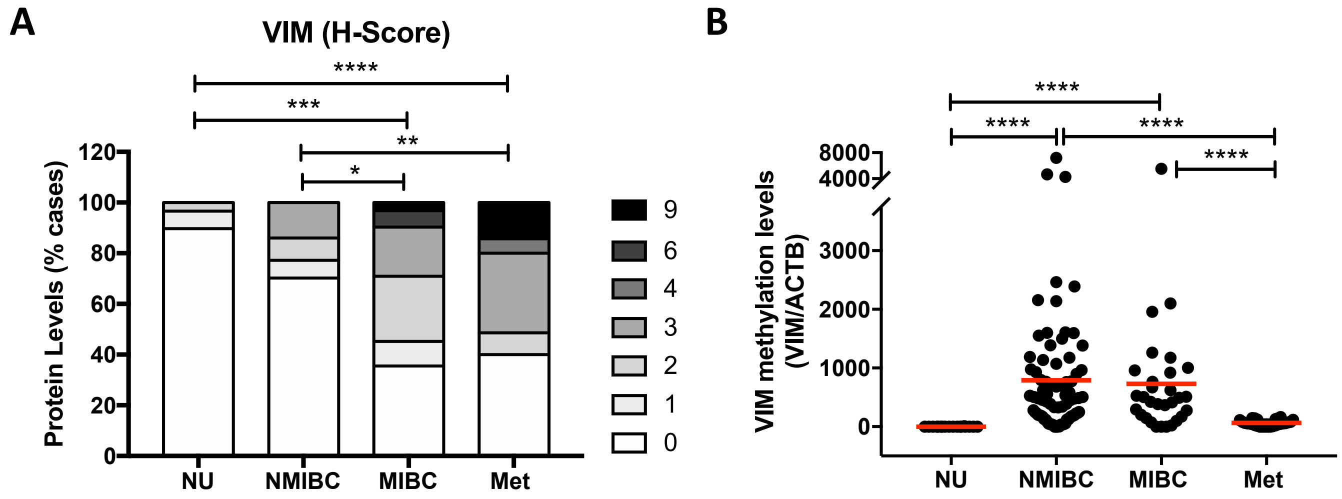
A
VIM (H-Score)
****
***
**
*
Protein Levels (% cases)
120
100
80
60
40
20
0
NU
NMIBC
MIBC
Met
9
6
4
3
2
1
0
B
****
****
****
****
8000
4000
3000
2000
1000
0
VIM methylation levels (VIM/ACTB)
NU
NMIBC
MIBC
Met

Figure S2

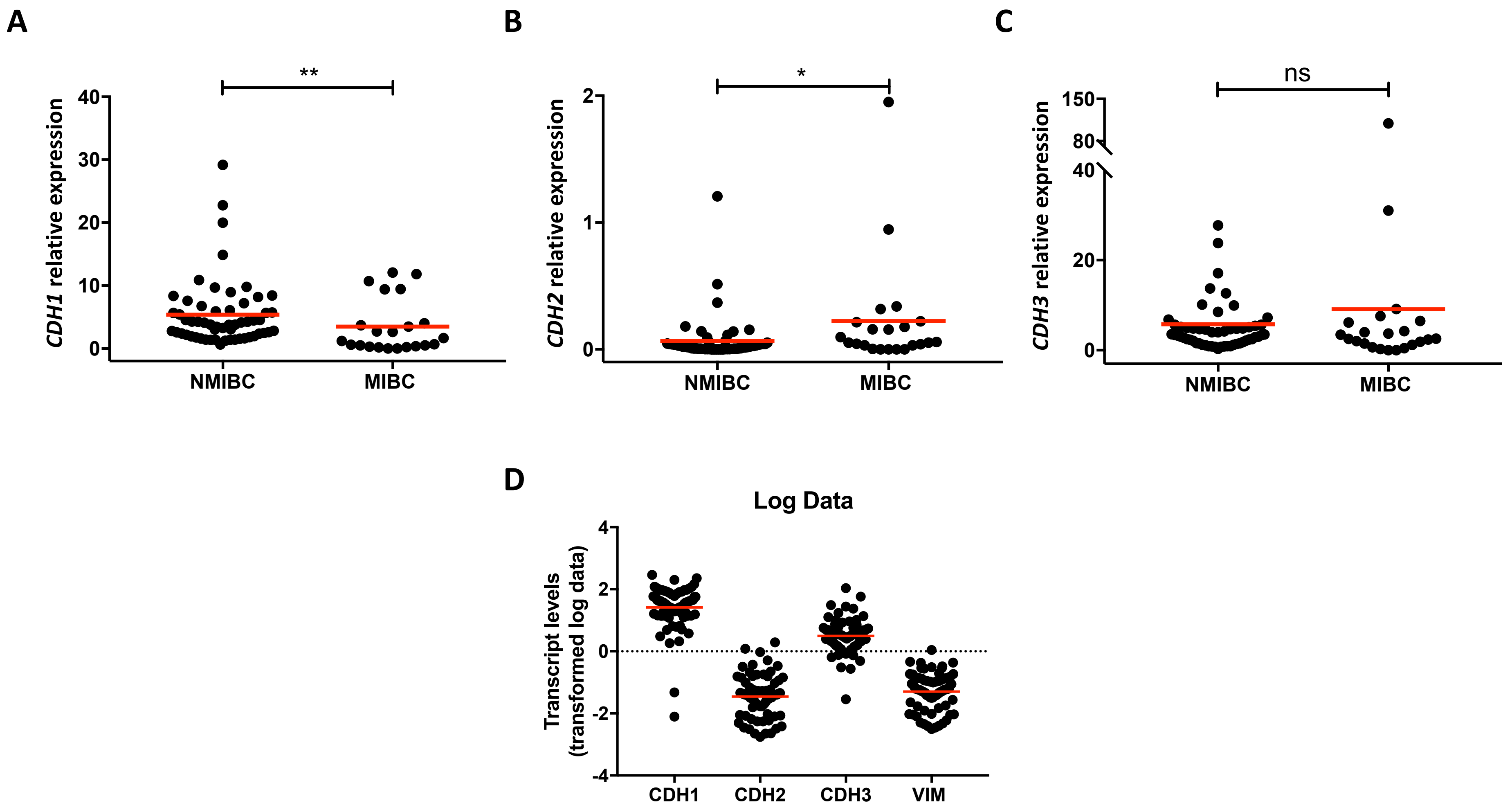
A
B
C
D
CDH1 relative expression
CDH2 relative expression
CDH3 relative expression
NMIBC
MIBC
**
*
ns
Log Data
Transcript levels (transformed log data)
CDH1
CDH2
CDH3
VIM

Figure S3

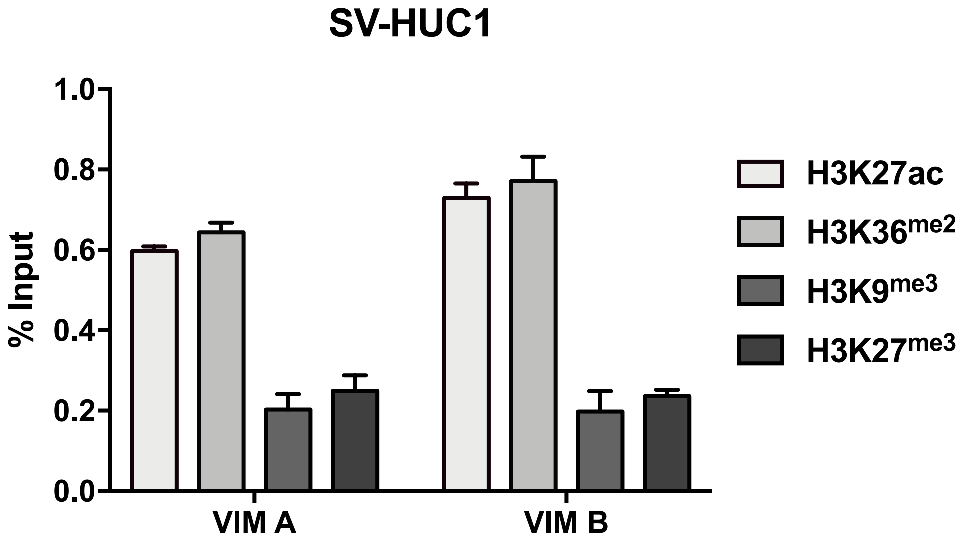
SV-HUC1
1.0
0.8
0.6
0.4
0.2
0.0
% Input
VIM A
VIM B
H3K27ac
H3K36me2
H3K9me3
H3K27me3

Table S1: Spearman correlation between *CDH1*, *CDH2*, *CDH3* and *VIM* transcript levels in Bladder cancer tissues. (Grey results are non-significant)

**Spearman r**	***CDH1***	***CDH2***	***CDH3***	***VIM***
CDH1		-0.224	0.212	-0.367
CDH2	-0.224		0.149	0.386
CDH3	0.212	0.149		-0.108
VIM	-0.367	0.386	-0.108	

Table S2: Primer sequences used in qMSP and RT-qPCR for studied genes.

Target Gene		Sequence (5'- 3')	$T_{Annealing}$ ºC
**Quantitative Methylation Specific PCR**			
**ACTB**	**Forward Primer**	TGGTGATGGAGGAGGTTTAGTAAGT	60
	**Reverse Primer**	AACCAATAAAACCTACTCCTCCCTTAA	
	**Probe**	FAM - ACCACCACCCAACACACAATAACAAACACA - MGB	
**VIM**	**Forward Primer**	TTCGGGAGTTAGTTCGCGTT	60
	**Reverse Primer**	ACCGCCGAACATCCTACGA	
	**Probe**	FAM - TCGTCGTTTAGGTTATCGT - MGB	
**Real-Time Quantitative PCR**			
**CDH1**	**Forward Primer**	CTTTGACGCCGAGAGCTACA	64
	**Reverse Primer**	AAATTCACTCTGCCCAGGACG	
**CDH2**	**Forward Primer**	TGTATGTTTTCCTTTCAGTGAATCTT	60
	**Reverse Primer**	TGGAAAGCTTCTCACGGCAT	
**CDH3**	**Forward Primer**	ACGAAGACACAAGAGAGATTGG	60
	**Reverse Primer**	CGATGATGGAGATGTTCATGG	
**HPRT1**	**Forward Primer**	TGACACTGGCAAAACAATGCAGACTT	60
	**Reverse Primer**	TTCGTGGGGTCCTTTTCACCAGCAA	

Table S3: References of antibodies used in ChIP assays for studied histones and histones posttranslational modifications.

Histone/ Histone PTM	Antibody Reference
**H3**	ab1791 (Abcam)
**AcH3**	06-599 (Millipore)
**H3K4me3**	ab8580 (Abcam)
**H3K9Ac**	17-658 (Millipore)
**H3K9me3**	07-442 (Millipore)
**H3K27me3**	07-499 (Millipore)
**H3K27Ac**	ab4729 (Abcam)
**H3K36me2**	ab9049 (Abcam)
**H4**	ab70701 (Abcam)
**H4K20me3**	ab9053 (Abcam)
